# A Standardised Exercise Protocol to Induce Oxidative Stress in Humans: Validation with a Dietary Polyphenol Intervention

**DOI:** 10.3390/nu17182971

**Published:** 2025-09-16

**Authors:** Oiva V. Kamati, Laura Bragagna, Dirk J. Bester, Karl-Heinz Wagner, Vera Stürmer, Markus Gassner, Lina Maqboul, Roan Louw, Sacha West, Simeon Davies, Jeanine L. Marnewick

**Affiliations:** 1 Applied Microbial and Health Biotechnology Institute (AMHBI), Cape Peninsula University of Technology, Cape Town 7540, South Africa; oivaviety@gmail.com (O.V.K.); daviess@cput.ac.za (S.D.); 2 Department of Nutritional Sciences, University of Vienna, 1090 Vienna, Austria; laura.bragagna@univie.ac.at (L.B.); karl-heinz.wagner@univie.ac.at (K.-H.W.); stuermervera@yahoo.de (V.S.); markus.gassner@univie.ac.at (M.G.); lina.maqboul@univie.ac.at (L.M.); 3 Vienna Doctoral School of Pharmaceutical, Nutritional and Sport Sciences, University of Vienna, 1090 Vienna, Austria; 4 Faculty of Health and Wellness Sciences, Cape Peninsula University of Technology, Cape Town 7540, South Africa; besterd@cput.ac.za; 5 Human Metabolomics, Faculty of Natural and Agricultural Sciences, North-West University, Potchefstroom Campus, Potchefstroom 2520, South Africa; roan.louw@nwu.ac.za; 6 Centre for Sport Business and Technology Research (CSBTR), Cape Peninsula University of Technology, Newlands Cricket Ground, Campground Rd, Cape Town 7700, South Africa; wests@cput.ac.za

**Keywords:** oxidative stress model, exercise, rooibos, dietary polyphenolic intervention

## Abstract

**Background:** Generating oxidative stress in a predictable and controllable way without the risk of causing harm is important for enabling the safe validation of interventions such as dietary polyphenols and ensuring ethical standards in human studies, while also advancing mechanisms involved in the induced oxidative stress. Although, many experimental animal and in vitro models have been developed to conduct oxidative stress-based research, to date, very few reliable human models are available. **Objective:** This study’s main objective was to establish a standardised exercise model to induce oxidative stress in a repeatable and controllable manner and was tested with dietary polyphenols. **Method:** We applied a single blinded, randomised, cross-over, placebo-controlled trial with adult (25.95 ± 6.25 years) males (N = 40) where the induction of oxidative stress was achieved by an incremental aerobic exercise activity followed by a maximal anaerobic activity until exhaustion. To assess the model, rooibos polyphenolics was used as one of the interventions, while markers of safety and oxidative stress were measured on various occasions during the trial period. **Results:** The exercise regime reliably and repeatably induced oxidative stress, evidenced by increased levels of oxidative damage markers, i.e., oxidised glutathione (*p* = 0.003), malondialdehyde (*p* = 0.004), and a Comet assay tail moment (*p* < 0.05), while unconjugated bilirubin (*p* = 0.002) and the ferric reducing antioxidant potential (*p* < 0.001) increased over the study period, in the male study participants, irrespective of the oral intervention. **Conclusions:** This model showed an exercise regime that could be adapted to induce oxidative stress in a reliable and repeatable fashion without risk of causing harm. This study also demonstrated that a dietary polyphenolic intervention with rooibos did not complicate the onset of oxidative stress.

## 1. Introduction

Oxidative stress was conceptualised several decades ago and defined as the imbalance of pro-oxidative molecules and antioxidant molecules within a system [1]. The role of free radicals and antioxidants is still not fully understood, but it is known that radicals have several signalling and inflammatory functions within the body [2,3]. Within these contexts, free radicals may, in general, also be seen as beneficial mediators; however, during pathology and with ageing, the natural antioxidant defence systems which regulate the levels and activity of many radicals are frequently overwhelmed, becoming inefficient, which results in tissue damage [4,5]. Thus, oxidative stress is seen to contribute to the pathophysiology of several diseases, and in some cases, to be involved in the aetiology thereof [2].

Despite this consensus on the importance of oxidative stress for human health, it is widely acknowledged that this phenomenon is not yet well enough understood to allow for effective clinical application [2,3]. One of the main reasons why there is a relative lack of knowledge regarding oxidative stress and the natural role of radicals and antioxidants may be the great biological diversity exhibited both in and between species regarding these mediators [6]. These same authors further expand on how several body systems, like the respiratory, digestive, cardiovascular, and endocrine systems, and lifestyle features, including dietary patterns, physical activity, and environmental exposure, significantly impact the expression of antioxidant defences and the generation of free radicals. Environmental factors including exposure to exogenous (environmental and synthetic) and endogenous (natural) toxic agents influence the antioxidant defence system and contribute to the formation or exacerbation of the generation of free radicals [7]. Antioxidant deficiencies expressed in individuals often develop because of decreased antioxidant intake, decreased efficiency of the endogenous antioxidant system due to disease, ageing, or increased antioxidant utilisation [8]. Since, free radicals are generated as by-products of normal cellular metabolism, the exposure of humans and other living organisms to radicals is unavoidable. However, the extent of damage incurred from the production and/or accumulation of free radicals depends on the nature of the targeted macromolecule, concentration and location of the targeted macromolecule, and occurrence of secondary damaging events such as chain reactions [9]. This lack of predictability and controllability of the free radical production and antioxidant expression may have contributed to the general lack of human models that are suitable for conducting human intervention studies.

It is evident from the literature that there are many in vitro and animal models which have been developed over the years to conduct oxidative stress-based research. A number of human studies have also been reported on using different exercise regimes to induce oxidative stress. However, recent systematic reviews indicated that differing modes, durations, and intensities of exercise/physical activity used in these studies may result in either increased or decreased oxidative damage, and therefore are characterised by inconsistency, implying that more studies should be carried out to provide a better and more comprehensive stress model, with a defined protocol when researching potential herbal interventions for reducing oxidative stress-induced damage [10,11].

Much of the experience gained from previous work with athletes may be used to inform the development of a controlled human oxidative stress model. The so-called incremental or ramp test coupled with breath-by-breath ventilation and gas exchange measurements is becoming one of the most powerful paradigms or models for experimental (sport and exercise) and clinical cardiopulmonary assessment. Thus, it is reasonable to assert that sport and exercise researchers have pioneered cardiopulmonary exercise testing to measure exercise capacity, which provides guidance in the differential diagnosis of exercise limitations [12], and clinicians have used exercise stress testing as a typical tool for diagnosing coronary artery disease or determining exercise capacity [13].

The justification for this paper pivots on the observation that less consideration has been given to a recognised exercise-induced oxidative stress model in humans, and as a consequence, there is no commonly recognised exercise protocol and/or model to dependably enact such an assessment. The objective of this study was premised on the absence of a standardised and reproducible exercise test to consistently induce oxidative stress, and overcoming this shortcoming, the present paper proposes such a replicable and valid model. The general hypothesis for this paper contends that the controlled human oxidative stress model described in this paper will provide researchers with an appropriate, reliable, and valid exercise test to consistently induce the production of excess reactive oxygen species during experimental studies. The present study took into account that most of the human daily activity is of low intensity (aerobic), punctuated by bouts of higher-intensity activity. It is reasonable to assert that humans are well suited to this mix of low-intensity physical activity interspersed with bouts of high intensity. In summary the daily physical exercise regimen that informs the aerobic–anaerobic exercise model to induce oxidative stress therefore provides the rationale for this paper and is aimed at developing an exercise-induced oxidative stress model in healthy humans, suitable for testing dietary interventions.

## 2. Materials and Methods

### 2.1. Study Design and Sample Size

**Figure 1:**
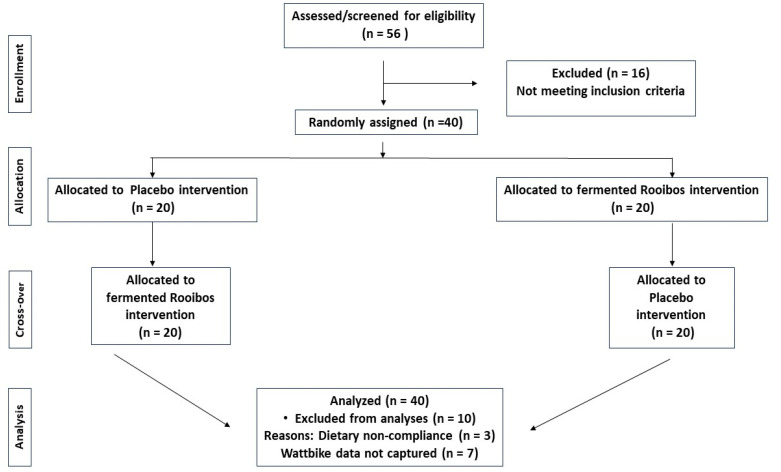
Consolidated Standards of Reporting Trials (CONSORT) flow diagram for this study.

The proposed model was implemented as a randomised, single-blinded, placebo-controlled, cross-over dietary intervention trial. This intervention refers to the ingestion of a standardised fermented rooibos beverage (375 mL), or a placebo beverage together with a standardised snack (sandwich), before being subjected to a regimen of exercise. Each participant attended two sessions with a seven-day washout period separating the cross-over between placebo and rooibos interventions (Figure 1), allowing the same participants to contribute to the control and experimental interventions randomly allocated in these two sessions. The exercise regimen used in this model consisted of an incremental aerobic exercise test phase that is frequently referred to as a modified submaximal ramp test, followed by a maximal anaerobic phase including 10 sets of sprints (or up until voluntary withdrawal) on a stationary Wattbike cycle ergometer.

The sample size was determined using G-Power 3.1 with the following input: tail(s) = one; effect size dz = 0.3; α err prob = 0.05; power (1 − β err prob) = 0.8, and following output: non-centrality parameter δ = 2.5278449; critical t = 1.6669145; *df* = 70; total sample size = 71; actual power = 0.8046743. More participants (*n* = 40) to complete the cross-over study were recruited to account for any loss during the study period.

### 2.2. Participant Screening and Recruitment

Participant recruitment took place after ethical clearance for this study was obtained from the Faculty of Health and Wellness Sciences Human Research Ethics Committee, Cape Peninsula University of Technology (project registration number: CPUT/HWS-REC 2018/H2). The ethical guidelines followed in this study were in accordance with the Declaration of Helsinki. As such, written informed consent was provided by each study participant before starting the exercise trial regime. This study adhered to the Consolidated Standards of Reporting Trials 2010 (CONSORT) guidelines and the CONSORT checklist.

Potential participants were then recruited by posting study leaflets at the study site (Human Performance Laboratory [HPL], CPUT, Mowbray campus, Cape Town) as well as by distributing leaflets to various sports clubs in the Cape Town area. Interested volunteers were asked to contact the principal investigator to be screened for study eligibility and completion of demographic and health and fitness history/information. Due to physiological and performance differences in exercise capacity and hormonal response to exercise between males and females [11], only adult males (18–50 years old) were recruited for this study. Inclusion criteria included the following: physically active male with no current or past history of (1) anabolic steroid use, (2) life-sustaining medication use for any chronic illness (hypertension, hyperglycaemia, hypercholesterolemia, thyroid or any metabolic disorders), (3) anabolic or ergogenic nutritional/antioxidant supplement use, (4) alcohol consumption of >2 beverages per day, (5) distinct dietary habits (diets other than omnivorous), and (6) sustained any muscular or skeletal injury in previous two months before the study. Additionally, participants were screened by fingerpick blood testing to ascertain that all values were within the normal reference ranges for the following parameters: total cholesterol (3.5–5.0 mmol/L or 135–193 mg/dL); fasting glucose (5.6–7.0 mmol/L or 100–126 mg/dL); haemoglobin (13.3–17.2 g/dL); and blood pressure (120/80 mmHg–<140/90 mmHg), as per reference ranges (mmol/L) published on the Pathcare Laboratory (South Africa) website. Those who met the inclusion criteria (*n* = 40) were then enrolled in this study after giving written informed consent. Anthropometric measurements were taken during the screening phase (baseline data) and the trial’s intervention phases. Blood pressure (systolic and diastolic) and other health indicators were also measured on these three occasions. Through completion of a health questionnaire, all study participants indicated that they engaged in physical activity for more than 30 min most days of the week, at moderate to vigorous intensity levels.

### 2.3. Randomisation and Controls

The study coordinator assigned each participant a unique study code, and as such, none of the data collected contained any direct identifiers of the participants. The main control for this study intervention was achieved by randomly dividing enrolled study participants into two intervention groups using a computer program randomizer.org (https://randomizer.org/). This was performed by the study coordinator. One group first received the rooibos intervention and the other the placebo intervention, while after a seven-day washout period, the groups were reversed. Participants were also required to keep a dietary record of all food and beverages they consumed for 3 non-consecutive days that consisted of 2 weekdays and 1 weekend day the week before they took part in the trial sessions. A food and beverage list of items high in antioxidant capacity and content was given to all participants to ensure restricted or limited intake of these items during the trial period. These items included an avoidance of coffee (all brands: pure and instant; caffeinated and decaffeinated; filter, percolated or plunger), tea (all brands: black, green, herbal, ice, flavoured, and unflavoured), cocoa drinks (all brands, e.g., hot chocolate, Milo, Ovaltine), red and rosé wines (all labels), fruit juices (100% pure juices and blends of all brands of red grape, orange, apple, and berry juices), and a limited intake of soda drinks (one can/340 mL), white wine (two glasses with 120 mL per glass), beer (340 mL), and spirit drinks (25 mL). In addition, the portions of the following fruits consumed daily were limited, apples (one daily), oranges or clementines (one/day), and black and red grapes/berries (one cup a week), while dark chocolate was restricted to three 40 g portions per week. Participants were further reminded not to change their daily dietary habits or physical activity throughout this study and not to take part in any physical activity or consume alcohol 24 h before their respective trial sessions.

### 2.4. Intervention Beverages

The intervention beverages (placebo and rooibos) were freshly prepared every day before the exercise session started. A commercially available peach and apricot-flavoured powdered beverage (375 mL) served as the placebo beverage. For the rooibos beverage, 1.6 g of a commercially available (Rooibos Ltd., Clanwilliam, South Africa), standardised, cold water-soluble fermented rooibos powdered extract was dissolved in 375 mL of the placebo beverage. Although nothing tastes like rooibos, the peach and apricot flavour overwhelmed that of rooibos and was found best suited for use as the placebo beverage. For blinding purposes of the participants and other team members, the principal investigator served both beverages in an opaque water bottle to disguise any colour differences there may have been. Both intervention beverages were characterised using high-performance liquid chromatography (HPLC) analysis as previously described by Bramati et al. [14]. Briefly, this entailed the use of an Agilent Technologies 1200 series system HPLC consisting of a quaternary pump, an autosampler, a degasser, a diode array, and a multiple-wavelength detector. A 5 µm (150 mm × 4.6 mm i.d.) column was used for the separation, and acquisition was set at 287 nm for aspalathin and at 360 nm for other major rooibos components. Water (solvent A) and methanol (solvent B) with 300 µL/L of trifluoroacetic acid in each solvent served as the mobile phases. The elution gradient started with solvent A at 95%, then 75% after 5 min and to 20% after 25 min and back to 95% after 28 min. The flow rate was set at 0.8 mL/min, with 20 µL as the injection volume and column temperature set at 23 °C. The retention times of the standard individual phenolics were utilised to identify the peaks, and concentrations were calculated using the standard area and sample area. Results were reported as mg/375 mL of beverage (Table 1). Only the rooibos beverage contained phenolic constituents when compared to the placebo beverage (HPLC chromatograms included as Supplementary Materials). Spectrophotometric methods were used to determine their antioxidant capacity, the total polyphenol content, as well as to identify the major polyphenolic constituents present (Table 1).

### 2.5. Trial Exercise Protocol

After an overnight fast of a minimum of eight hours, participants reported to the study site (HPL) and were given a light snack (162.8 g) consisting of two slices of white bread topped with a thin layer of margarine, pastrami, and cheese, and consumed the freshly prepared 375 mL of either the rooibos or placebo beverage. Ninety minutes thereafter, study participants commenced with the modified Wattbike submaximal ramp test protocol as described below.

#### 2.5.1. Warm-Up

- A 5 min warm-up, at 50–60 rpm on air resistance setting 3 on the Wattbike Pro

#### 2.5.2. Exercise Test Protocol to Induce Oxidative Stress

- Pedal in a seated position for 1 min at the starting power of 100 watts (W), at a cadence rate of between 70 and 100 rpm.
- Increase the air resistance setting and/or cadence as necessary every 1 min to ensure a 15 W increase in power (W) output every 1 min; this allowed the body to adapt to the increasing workload and for a steady heart rate to be achieved (at that level).
- Keep increasing the power (W) output by 15 W every minute until the rider reaches the rate of perceived exertion (RPE) of somewhat hard—level 13 on the Borg scale rate of perceived exertion.
- The test would be terminated if the rider/participant experienced any adverse symptoms during the exercise test prior to achieving their sub-maximum exertion of 75–80% as per heart rate and/or the participant rating the exertion level, as per the Borg scale at 13 (somewhat hard).
- The test would also be terminated if the participant experienced an emergency (physical and/or mental) e.g., the participant felt unwell and/or became overly anxious.
- Once the participant reaches level 13 on the Borg scale, he stops cycling for 1 min and then is asked to complete sets of 10 s sprints (10 max or until the participant reaches the RPE of maximal exertion—level 20 on the Borg scale), separated by 15 s of passive recovery rest periods.

The participants returned to the human performance laboratory seven days after completion of their first intervention and exercise session for the cross-over leg of this study.

### 2.6. Blood Sampling and Processing

Blood was drawn from the participant’s antecubital vein into serum separate (SST) and ethylenediaminetetraacetic acid (EDTA) tubes at five different time points during each trial intervention session. These times include a fasting/baseline sample, indicated as 0 h, a sample taken 90 min after ingestion of the study beverage and snack before the exercise regime commenced (indicated as 1.5 h), immediately after the exercise regime was completed (indicated as IAE), then again 1 h post exercise regime (indicated as 1 h rest), and the final fasting sample drawn 24 h post exercise regime (indicated as 24 h rest). All blood tubes were inverted a few times after collection and stored on ice before processing. For the EDTA blood, 100 μL was aliquoted into a 2 mL Eppendorf tube containing 10 μL of methyl-2-vinylpyridinium (M2VP) for analysis of oxidised glutathione (GSSG), and another 50 μL was aliquoted into a 2 mL Eppendorf tube for reduced glutathione (GSH) analysis. The remaining EDTA blood was centrifuged at 2000 rpm and 4 °C for 10 min, whereafter the plasma, buffy coat, and red blood cells were aliquoted into different 2 mL Eppendorf tubes for storage. The SST blood samples were first allowed to clot before being centrifuged to obtain serum aliquots. All sample aliquots were stored at −80 °C until the time of analysis.

### 2.7. Oxidative Stress Markers

In this study, the exercise regime was intended to induce oxidative stress in a predictable and controllable manner to establish a physiological oxidative stress model. Other lifestyle factors which may give rise to oxidative stress, such as diet, disease conditions, and drug usage, were controlled as previously described.

#### 2.7.1. Reduced Glutathione

Glutathione (GSH) serves as an endogenous low-molecular-weight antioxidant and forms an important part of the endogenous antioxidant defence system. This study used the GSH recycling assay based on the enzymatic recycling of GSH in the presence of glutathione reductase (GR) as described by Asensi et al. [15] to determine the total glutathione content of whole blood samples collected. Briefly, 75 mM of phosphate buffer (pH 7.4) was prepared by dissolving 10.36 g of sodium dihydrogen monophosphate (NaH_2_PO_4_·H_2_O, Sigma-Aldrich, Johannesburg, South Africa) in 1 L of double-distilled water (DDW), while 3 mM of GSH (0.046 g of GSH standard, Merck, Johannesburg, South Africa, dissolved in 50 mL of buffer) was used to make a 3 µM GSH stock solution, used to prepare the standard series (0.5, 1.0, 1.5, 2.0, 2.5 µM). The assay enzyme was prepared by diluting 80 µL of GR (168 U/mg, Merck, Johannesburg, South Africa) with 4920 µL of buffer, while 6 mg of 5,5′-dithiobis-2-nitrobenzoic acid (DTNB) was dissolved in 50 mL of buffer. A 5% meta-phosphoric acid (MPA) solution was prepared by dissolving 2.5 g of MPA in 50 mL of DDW, whereas 12 mL of buffer was added to 10 mg of nicotinamide adenine dinucleotide phosphate hydrogen (NADPH) to yield a concentration of 0.83 mg/mL. One 50 µL whole-blood sample aliquot was thawed and equilibrated to room temperature (RT). Thereafter, 350 µL of 5% MPA was added to the sample tube, vortexed for 15–20 s, and centrifuged (Eppendorf 5810R, Eppendorf South Africa (Pty) Ltd., Johannesburg, South Africa) at 14,000 rpm and 4 °C for 4 min. The supernatant (10 µL) was pipetted into a new 1.5 mL Eppendorf tube, mixed with 600 µL of buffer, and vortexed thoroughly. The assay was conducted in triplicate in a clear, 96-well microplate, with each well containing 50 µL of standard/participant sample, 50 µL of DTNB, and 50 µL of GR. To initiate the reaction, 50 µL of NADPH was added to each well, and the microplate was read immediately for 5 min in the SpectraMax i3x platform plate reader (Molecular Devices, San Jose, CA, USA) set at 25 °C and 412 nm. Data analyses were performed in the Microsoft^®^ Excel 2016 program; the tGSH concentration was calculated using the calibration curve of GSH at different concentrations, while the GSH concentration was calculated as per the formula below. The results were expressed as GSH concentration activity µmol/L blood.

GSH = (tGSH − (2 × GSSG))

#### 2.7.2. Oxidised Glutathione

The GSSG level was determined using a similar procedure as that described for the GSH quantification above. All reagents were freshly prepared exactly in the same way as in GSH determination. However, the GSSG standard (Merck, Johannesburg, South Africa) was used to make a 1.5 µM stock solution, from which the standard series (0.5, 1.0, 1.5, 2.0, 2.5 µM) were prepared. The M2VP-treated whole-blood sample aliquots were thawed and left at RT for 2–10 min, before 290 µL of 5% MPA was added, vortexed for 15–20 s, and centrifuged (Eppendorf 5810R, Johannesburg, South Africa) at 14,000 rpm and 4 °C for 4 min. We pipetted 25 µL of the supernatant into a new 1.5 mL Eppendorf tube, mixed it with 350 µL of a prepared buffer, and vortexed thoroughly. The assay was conducted in triplicate, and each well of a clear, 96-well microplate consisted of 50 µL of standard/participant sample, 50 µL of DTNB, and 50 µL of GR. The microplate was incubated at RT for five minutes, and then 50 µL of NADPH was added to each well (multi-channel pipette used) to start the reaction. The microplate was read immediately for 5 min in the SpectraMax i3x platform plate reader (Molecular Devices, San Jose, CA, USA) set at 25 °C and 412 nm. Data analyses were performed in the Microsoft^®^ Excel 2016 program, the GSH concentration was calculated using the calibration curve of GSH at a different concentration, and the results were expressed as the GSSG concentration activity µmol/L.

#### 2.7.3. The Redox Status of Glutathione

The GSH/GSSG ratio provides valuable information on the redox status of glutathione. In this study, the GSH/GSSG ratio was calculated according to the formula below:

GSH/GSSG ratio = GSH ÷ GSSG

#### 2.7.4. Conjugated Dienes

The conjugated diene (CD) is a primary product of lipid oxidation (LPO), resulting from the rearrangement of the carbon double bond when free radicals removed hydrogen atoms from a fatty acyl methylene group. In this study, the CD concentration was determined as per the procedure described by Recknagel et al. [16]. Briefly, plasma sample aliquots were thawed and equilibrated to RT, and then 100 µL was pipetted into a 2 mL Eppendorf tube, followed by 400 µL of chloroform and methanol mixture at a 2:1 ratio (Merck, Johannesburg, South Africa), vortexed for 10 s, and then centrifuged (Eppendorf 5810R, Johannesburg, South Africa) at 10,000 rpm and 4 °C for 10 min. Thereafter, 200 µL of the bottom layer (chloroform) of the tube was removed and pipetted into a new 2 mL Eppendorf tube, which was then left at 4 °C overnight with the lid open to allow the chloroform to evaporate. Thereafter, the dried sample was dissolved by adding 1000 µL of cyclohexane (Merck, Johannesburg, South Africa), vortexed for 10 s, and used as an assay sample. The assay was conducted in triplicate in a clear, 96-well microplate, with each well containing 300 µL of the participant sample. The absorbance was read against pure cyclohexane as a blank in the SpectraMax i3x platform plate reader (Molecular Devices, San Jose, CA, USA) set at 25 °C and 232 nm. Sample CD concentrations were calculated based on the difference in absorbance of the sample and the blank (calculation equation is shown below), and the results were reported as µmol/L plasma.

#### 2.7.5. Thiobarbituric Acid Reactive Substances (TBARSs)

The malondialdehyde (MDA) measured as TBARS is an indicator of LPO formed at the end stage when decomposition products of LPO, MDA, react with thiobarbituric acid (TBA). In this study, the TBARS assay was performed as described by Draper et al. [17] with slight modifications. All the reagents were freshly prepared. We prepared 4 mM of butylated hydroxytoluene (BHT, Merck, Johannesburg, South Africa) by dissolving 0.0088 g of BHT in 10 mL of ethanol (Sigma-Aldrich, Johannesburg, South Africa), while 0.1 M of sodium hydroxide solution (NaOH) was prepared by dissolving 0.2 g of NaOH in 50 mL of DDW. This was used to dissolve 0.159 g of TBA (Sigma-Aldrich, Johannesburg, South Africa) in a 10 mL tube. We added 684 µL of concentrated ortho-phosphoric acid (H_3_PO_4_) to 50 mL of DDW to make a 0.2 M orthophosphoric acid reagent. Plasma participant sample aliquots were thawed and equilibrated to RT, and 100 µL was pipetted into a 2 mL Eppendorf tube, followed by 12.5 µL of BHT, 100 µL of H_3_PO_4_, and 12.5 µL of TBA. The tube was vortexed for 10 s, and then a small hole was punched through the tube’s lid before it was placed in a heating block set at 90 °C for 45 min (MDA and TBA react and form a pink-coloured product). After heating, the tube was cooled immediately by placing it on ice (to stop the MDA-TBA reaction) for 2 min before 1000 µL of butanol (Merck, Johannesburg, South Africa) and 100 µL of saturated salt (sodium chloride) were added. The tube was vortexed again for 10 s, then centrifuged (Eppendorf 5810R, Johannesburg, South Africa) at 10,000 rpm and 4 °C for 2 min, and the supernatant was used as an assay sample. The assay was performed in triplicate in a clear, 96-well microplate, each well filled with 300 µL of the sample. The absorbance was read against a blank (butanol) in the SpectraMax i3x platform plate reader (Molecular Devices, San Jose, CA, USA) set at 25 °C and 532 nm. The concentration of plasma TBARS was calculated based on the difference in absorbance of the sample, and the blank results were reported as µmol/L plasma.

#### 2.7.6. Protein Carbonylation

Protein oxidation implies an irreversible modification/damage of proteins caused directly or indirectly by RONS or oxidative stress by-products. This study makes use of the procedure described by Colombo et al. [18]. All reagents were freshly prepared except the dinitrophenylhydrazine (DNPH) solution, which was prepared by dissolving 198 g of DNPH (Sigma-Aldrich, Johannesburg, South Africa) in 100 mL of 2.5 M HCl, left overnight in the dark at RT. We prepared 20% and 10% trichloro acetic acid (TCA) solutions by dissolving 20 g and 10 g, respectively, of 100% TCA (Sigma-Aldrich, Johannesburg, South Africa) in 100 mL of DDW. A 6 M guanidine hydrochloride solution was prepared by dissolving 27.32 g of 6 M guanidine hydrochloride (Whitehead Scientific, Cape Town, South Africa) in 100 mL of DDW, while 100 mL of 2.5 M HCl was prepared by adding 24.635 mL of 36.46 MW HCl (Merck, Johannesburg, South Africa) to 75.365 mL of DDW. Normalised participant protein sample aliquots (1 mg/mL) were thawed and equilibrated to RT, and then 200 µL was pipetted into a 15 mL Sterilin tube followed by 800 µL of 10 mM DNPH solution. A blank sample for each participant sample was prepared similarly by adding 200 µL of normalised sample aliquot to a 15 mL Sterilin tube, followed by 800 µL of 2.5 M HCl (without DNPH). The Sterilin sample tube was then incubated at RT for 60 min with vortex mixing every 10–15 min. Thereafter, 1 mL of 20% TCA was added to the Sterilin tubes (sample and blank tube), vortexed, and incubated on ice for 10 min. Thereafter, tubes were vortexed and centrifuged (Eppendorf 5810R, Johannesburg, South Africa) at 2000 rpm and 4 °C for 10 min. The supernatant was then discarded, and 1 mL of 10% TCA was added (to wash the pellets), vortexed, and centrifuged again. The supernatant was discarded before 1 mL of 1:1 (*v*/*v*) ethanol: ethyl acetate was added to further wash protein pellets. Centrifuging and addition of 1 mL of 1:1 (*v*/*v*) ethanol: ethyl acetate was repeated twice just to remove free DNPH debris and lipid contaminants (clear supernatant). After the last wash, pellets were dissolved with 500 µL of 6 M guanidine hydrochloride and incubated at 37 °C for 10 min. After incubation, dissolved sample protein pellets were vortexed and centrifuged (Eppendorf 5810R, Johannesburg, South Africa) at 2000× *g* and 4 °C for 10 min. The supernatant was pipetted to a clear, 96-well microplate, with 100 µL per well (in triplicate), and the absorbance was read in the SpectraMax i3x platform plate reader (Molecular Devices, San Jose, CA, USA) set at 25 °C and 370 nm. The samples’ PC content was calculated [19] and the results were reported as µmol/L plasma.

#### 2.7.7. Unconjugated Bilirubin

Unconjugated bilirubin (UCB) is one of the most important endogenous, non-enzymatic antioxidants. It was determined by high-performance liquid chromatography (HPLC) as already described previously by Wallner et al. [20]. Serum obtained from the blood of the subjects was used for the analyses, where 50 µL of serum was mixed with 200 µL of mobile phase (consisting of 96.5% methanol and 3.5% distilled water) and centrifuged at 14,000 rpm for 10 min. Thereafter, 120 µL of supernatant was pipetted into vials and used for analysis. Serum UCB concentrations were measured by HPLC (Merck-Hitachi Lachrom, Gerbershausen, Germany), equipped with a Fortis C18 HPLC column (4.6 × 150 mm, 3 mm), Phenomenex Security Guard cartridges for C18 HPLC columns (4 × 3 mm), and a photodiode array detector (PDA, Shimadzu, Komeuburg, Austria). The bilirubin standard was from Sigma Aldrich (Vienna, Austria). The concentration was expressed in µmol/L.

#### 2.7.8. Ferric Reducing Antioxidant Power (FRAP) Assay

The FRAP assay is a spectrophotometric method for the determination of total antioxidants in plasma. For these analyses, the method of Benzie et al. was used [21]. Therefore, 10 µL of EDTA plasma was mixed with 30 µL of distilled water and 300 µL of FRAP reagent (50 mL of acetate buffer, 5 mL of TPTZ solution, 5 mL of ferric chloride hexahydrate reagent (Merck) on a 96-well microplate, incubated at 37 °C for 6 min, and then analysed at 540 nm. Using an FeSO_4_ standard (Merck), the concentration of total antioxidants was calculated and expressed in µmol/L.

#### 2.7.9. Oxidative DNA Damage

The Comet assay is a method for visualising DNA damage in single cells. The protocol was performed as described by Draxler et al. [22]. In short, 10 µL of whole-blood samples was mixed with 200 µL of 1% agarose solution, and 5 µL of the mixture was spotted onto 12 spot microscopy slides. For every blood sample, 4 slides were needed due to the 4 different treatments. To purify the DNA of the blood samples from cell debris, all microscopy slides were treated with a lysis buffer for one hour. Two of the microscopy slides were washed 3 times with enzyme buffer (40 mM HEPES, 0.1 M KCL, 0.5 mM EDTA, 0.2 mg/mL BSA, pH 8). On each spot of one of the two slides, 30 μL of the lesion-specific enzyme formamidopyrimidine DNA glycosylase (FPG) (diluted 1:3500 in enzyme buffer) was applied, using a slide unit (12-Gel Comet Assay Unit™, Severn Biotech Limited, Kidderminster, UK). On the second slide, 30 μL of the enzyme buffer alone was applied. Both slides were incubated at 37 °C for 30 min. Treatment with enzyme buffer alone acted as a blank for the FPG treatment. The fourth slide was incubated for 15 min with the oxidising agent H_2_O_2_ (100 μM), which allowed the indirect measurement of antioxidant status and represented resistance to H_2_O_2_. After the treatment steps, the DNA on the microscopy slides had to unwind in an alkaline electrophoresis buffer for 20 min (0.3 M NaOH, 1 mM EDTA, pH 13), followed by a 30 min electrophoresis run (25 V, 300 mA, 4 °C). The extent of DNA damage could then be visualised and evaluated by colouring the DNA with GelRed (GelRed TM Nucleic Acid Gel Stain, VWR, product no. 41003) and using a fluorescence microscope (Nikon Europe B.V., Amstelveen, The Netherlands). The software used to accurately determine DNA damage was Comet Assay IV.

### 2.8. Clinical Chemistry Markers

Various serum chemistry analytes as indicators of liver and kidney function were measured using commercially obtained Medica EasyRA kits and the clinical chemistry automated analyser, Medica Easy Random Access (EasyRA, Bedford, MA, USA). All kits and reagents were purchased from Medical Electronic Distributors (Cape Town, South Africa) and stored at 2–8 °C. Briefly, the analyser uses a wireless radio frequency identification technology to manage the utilisation and protocols for up to 24 on-board reagents packaged in liquid form in single or dual reagent wedges ready to use. Participant serum samples were thawed at RT, and analysis was carried out in duplicate. Calibration was performed on a regular basis while internal quality controls were run every day before participant sample analysis.

### 2.9. Statistical Analysis

Statistical analyses were performed using IBM SPSS Statistics 28 software. After testing for a normal distribution using a Shapiro test, a repeated-measures analysis of variance (RM-ANOVA) was used for statistical evaluation of DNA damage and oxidative stress markers across the 5 time points and a comparison of both groups, placebo and rooibos. The Greenhouse–Geisser adjustment was used to correct for violations of sphericity. A level of *p* < 0.05 was considered statistically significant. Bonferroni-adjusted post hoc analysis was then performed for all individual measurement time points. Microsoft 365 Excel was used to determine the percentage changes in values between the two points in time.

## 3. Results

### 3.1. Anthropometric Measurements and Health Indicators

Anthropometric measurements, blood pressure, and certain health indicators of the study participants (*n* = 30) during each of the study intervention sessions are indicated in Table 2. The data of ten (10) participants had to be excluded from the final dataset because of incomplete Wattbike data captured and non-compliance with the dietary requirements and/or use of prescription drugs noted after assessment of the dietary records.

### 3.2. Oxidative Stress Parameters

#### 3.2.1. Glutathione Redox Status

When considering the circulating total glutathione (tGSH) and reduced glutathione (GSH) levels, a significant time effect (*p* < 0.001) and moderate (*p* = 0.055 and *p* = 0.063, respectively) time–group effect were evident with both study interventions, but only rooibos resulted in a sustained increased glutathione level up to 24 h after completion of the exercise regime (Table 3). For the oxidised glutathione (GSSG) levels, a significant time effect (*p* = 0.003) was shown for both interventions, with a sharp increase immediately after the exercise regime was completed and a decrease to baseline levels and lower following the 1 h and 24 h rest periods. No differences (*p* = 0.436) were shown when comparing the two study interventions here. As a result of the effects on the reduced and oxidised glutathione levels, the glutathione redox status ratio (GSH:GSSG) was significantly (*p* = 0.003) changed by both study interventions, over time, but no differences were shown between the two intervention groups (Table 3).

#### 3.2.2. Oxidative Stress Status and Lipid and Protein Damage

Both interventions showed a significant (*p* = 0.004) time effect for the late oxidative lipid damage marker. Plasma MDA increased after completion of the exercise regime with a continued increase after the 24 h rest period, while the CDs were moderately (*p* = 0.051) increased only immediately after completion of the exercise and returned to baseline levels after rest. When considering the group and the group x time effects for both the rooibos and placebo interventions on these oxidative lipid damage biomarkers, no significant changes were observed. None of the interventions significantly (*p* > 0.05) altered the participants’ plasma circulating protein carbonyl levels (Table 4). When considering the two antioxidant markers, FRAP and UCB, both showed a significant time effect (*p* = 0.002 and *p* < 0.001, respectively), with an increase in the two markers over the course of this study, peaking immediately after completion of the exercise regime for UCB and at 1 h rest for FRAP. However, no significant effect between the two interventions could be shown for UCB and FRAP.

#### 3.2.3. Oxidative DNA Damage

The mean values of DNA damage measured by the Comet assay in participants’ whole-blood samples during this study are presented in Table 5. When DNA damage was measured by the standard treatment, results for both lysis and H_2_O_2_ treatments showed a significant gradual increase in DNA tail damage (*p* = 0.011 for lysis; *p* < 0.001 for H_2_O_2_, respectively) from 1.5 h, which kept increasing till 24 h post-exercise, irrespective of the intervention beverage consumed. Results for the DNA lesion-specific enzyme [formamidopyrimidine glycosylase (FPG)] treatment showed a sharp, significant increase in DNA damage at 1.5 h, which then decreased immediately after the exercise regime was completed, but then increased again after the 1 h and 24 h rest periods (*p* < 0.001). These results for all three treatments indicate the DNA damage that occurred during the exercise regime irrespective of the interventional beverage consumed, which is a confirmation that the model was able to induce oxidative stress in the participants.

### 3.3. Clinical Chemistry Markers

Throughout this study, no adverse effects were reported by any of the study participants. Additionally, serum analysis of the effects of the two intervention beverages on study participants’ liver and kidney markers showed no adverse effects. The only significant differences detected were time effects, specifically increased levels measured immediately after completion of the exercise regime, whereafter they all returned to levels similar to that of the baseline after a rest period. This may be indicative that the exercise regime was rigorous enough to temporary increase liver and kidney markers, potentially by inflammatory processes in the muscle tissues, together with the associated metabolic and excretion burdens (Table 6). When considering the glucose and lactate levels, a significant time effect (*p* < 0.001) included a decreased level immediately after completion of the exercise regime for glucose, while for lactate, there was a significant increase (*p* < 0.001) (baseline levels 2.8 ± 1.41 increasing to 12.43 ± 2.98 U/L), returning to baseline levels again after a 24 h rest period. No significant changes in CK levels were observed at the various study time points, while LDH showed a marginal increase immediately after completion of the exercise regime, although this was not significant (*p* = 0.063).

## 4. Discussion

As per the aim of this study, we have designed and conducted a randomised, single blinded, cross-over, placebo-controlled intervention trial, to develop an exercise-induced oxidative stress model for healthy adult males, and tested it with rooibos dietary polyphenolics. The first step was to design an exercise regime to induce oxidative stress that is repeatable and applicable to individuals of varying fitness levels without causing harm. One major advantage of using exercise-induced oxidative stress is that it is associated with less risk than oxidative stress induction by other processes that could be considered pathological [2]. To ensure similarity and repeatability between the two oral interventions, to test the stress model, this study was designed as a cross-over trial with a seven-day washout period in between. This meant that the same participants would partake in both interventions at random (blinded) and then crossed over to the other following a 7-day washout period. Other important factors to control for, such as general physical activity, dietary intake, medicinal or recreational drug use of participants prior to or during the trial, and any pathological or physiological processes which may contribute to oxidative stress [2], have been considered in our design.

As evidenced by the oxidative stress marker results of our study, it can be deduced that the participants in this study had oxidative stress levels induced by the exercise regime, irrespective of the intervention beverage consumed. The changes over time in oxidative stress markers were most clearly demonstrated immediately after exercise by the significant increase in lipid and DNA oxidation markers, accompanied by increased glutathione oxidation. Furthermore, an increase in the concentration of the two antioxidant markers, UCB and FRAP, was observed over the course of the exercise challenge, which peaked immediately after completion of the exercise regime in the case of UCB and one hour later in the case of FRAP. This effect, which is frequently observed in exercise challenges, can be explained by the release of antioxidants into the plasma after physical exertion as an acute response to oxidative stress [23]. Additionally, although not significant in most cases, a clear trend was observed for some oxidative stress markers modulated by the rooibos beverage intervention to return to their baseline levels more rapidly when compared to the placebo intervention. These results affirm that the exercise test protocol (modified submaximal ramp test) used in this study has indeed induced oxidative stress damage in participants and also indicates the potential to successfully assess oral interventions such as rooibos.

It is important to keep in mind that factors such as the formulation type, dosage, timing of consumption, and bioavailability of the rooibos actives may all influence these study outcomes. Very few studies have reported on the use of rooibos in human intervention trials, but the majority of them point to poor bioavailability of the rooibos constituents when consumed either as a herbal tea or as polyphenolic-rich extracts, with trace amounts of the rooibos flavonoids and its metabolites detected. One such study reported a recovery of only 0.35% of aspalathin in the urine during the first 5 h after consumption [24]. Chronic or acute consumption of rooibos can also influence the magnitude of the effect. Previously, Marnewick et al. showed that consuming 1200 mL of rooibos herbal tea daily for six weeks significantly improved various blood biomarkers, including the glutathione redox status, in adults at-risk for developing cardiovascular disease [25]. Villano and co-workers also showed an increased antioxidant status in the blood that peaked 1 h after consuming an acute dose of 500 mL of rooibos [26], while another study showed no change in the antioxidant capacity of the serum after an acute dose of 500 mL of rooibos [27].

A number of human studies reporting on the use of different exercise regimes or models to induce oxidative stress are apparent in the literature. A study by Escobar et al. [28] reported that a short-term training period, consisting of repeated sprints, by elite soccer players led to elevations in these oxidative stress markers (TBARS, PC, SOD, CAT), while ultra-endurance running increased similar markers (PC, TBARS, TAC, 8-OH-dG) instantaneously in trained, endurance athletes, but with no reported increase in these biomarkers (TBARS, PC, TAC) after the running exercise regime [29,30]. Additionally, a cross-over trial by Jówko and co-workers [31] observed a similar outcome to the current study, where male sprinters, after a repeated cycling sprint exercise regime, showed increased lipid peroxidation damage (MDA levels), during the placebo intervention. This increase was reversed with a green tea extract intervention. Ferreira and colleagues concluded that after a SAFT90 protocol (a soccer-specific aerobic field exercise test), a hydro-electrolyte beverage (whey permeate with the phenolic extract of jaboticaba peel) consumed by male participants attenuated markers of muscular and oxidative stress damage (serum AST, ALT, and CK levels) in these trained individuals [32]. These studies all concluded that additional research is required to provide a better and more comprehensive stress model, with a defined protocol when researching potential herbal interventions for reducing oxidative stress-induced damage. Recent systematic reviews revealed that a large portion of inconsistency in oxidative stress human studies is primarily due to the type, modes, durations, intensities, and physical fitness of individuals as well as the types of blood markers that were analysed [33,34].

Based on our findings, the number of sprints completed by participants varies, and this may have impacted how oxidative stress was induced by the exercise regime. This observation is important because the variability of results generated in this study appears to have been affected by the training status and/or fitness level of the participants, as determined by the number of sprints they were able to complete. This outcome suggests inconsistencies in findings that emerged from this study and those previously conducted using both anaerobic and aerobic exercises can be attributed not only to the training status of participants but also due to large inter-individual variability in response to exercise-induced oxidative stress [35]. Additional factors that intersect with training status are anthropometry and associated morphological characteristics such as BMI; simply put, people come in different shapes and sizes, and this will invariably influence the relationship between exercise performance, exercise status, and the degree of oxidative stress experienced. Furthermore, the ability to tolerate oxidative stress during exercise may vary between the sexes and is likely to be influenced by genetics [36]. The same author noted that existing studies in humans have centred on young male adults, suggesting that future studies should be extended to children, women, and the aging population. This is because within all populations, there may be a genetic susceptibility to oxidative stress that is potentiated by certain types of exercise. Thus, it needs to be acknowledged that a standardised exercise protocol to induce oxidative stress in humans would better equip researchers with a reliable and consistent model for application with specific populations, as alluded to by Jenkins [36].

Apart from the physical fitness of participants, many human and animal studies indicated that the relationship between exercise and free radical production or extent of oxidative stress is a complex one and often dictated by duration, intensity, mode, and type of exercise [11,37]. Generally, most free radicals are generated by the mitochondria as a by-product of incomplete oxygen metabolism during cellular respiration [38]. During exercise, oxygen uptake increases due to the high energy demand, with about a 10- to 20-fold oxygen flux increase in the active skeletal muscles [39]. It has also been shown that during exercise, not all of the oxygen consumed by cellular mitochondria is converted to water; although the exact amount is not clear, a small percentage of consumed oxygen is instead reduced to free radicals such as superoxide anion (O^2−^), which subsequently is further reduced to the more potent hydroxyl radical (OH) [40,41]. A study by Richardson et al. concluded that prolonged or intense muscle contractions alter the physiological environment in the muscle fibres and predispose them to a higher rate of radical generation [42]. In 2004, Arbogast et al. reported that muscle contraction is associated with an increased muscle temperature, increased carbon dioxide tension, and decreased cellular pH, which may stimulate and accelerate free radical production within the muscles [43]. Similarly, Ferreira et al. also reported that increased oxygen consumption in muscle fibre during exercise lowered oxygen intracellular tension and augmented the generation of radicals [44].

## 5. Study Limitations

In this study, through the enrolment parameters, we sought to recruit a homogenous cohort of participants of healthy physically active males, which included inclusion criteria based on anthropometric measures, namely, stature, mass, and waist circumference, as well as BMI. These criteria were complimented by the assessment of total cholesterol, fasting glucose, haemoglobin, and blood pressure in terms of recognised reference ranges, as well as self-reported dietary and supplement intake and habitual exercise routine. However, whilst the study design sought to recruit a group of participants with similar characteristics, there may have been variability in their physical fitness levels, which could have affected their performance in the exercise test. It is acknowledged that this might be a confounder to the results of this study, given that the production of waste products and radicals may differ in individuals of varying levels of fitness [45]. It is thus recommended in future that when participants are enrolled, a control mechanism be introduced to ensure they are of similar fitness, or participants of different levels of fitness can be allocated into different experimental groups.

Another limitation to be considered is the applicability of the current study results to female participants. It is crucial to recognise that sex differences will influence the results, and future research should be tailored to the needs of women. A number of differences between the sexes, such as metabolic differences in fuel storage and utilisation, and understanding the role of hormonal fluctuations and oral contraceptives are important when planning exercise and nutrition experimental studies. A recent, comprehensive review addresses these important sex differences in physiological and anatomical systems and their effects on human physical performance [46].

Although the study participant population, healthy, young men, was selected to represent a relative homogenous study group, it is recognised that in order to optimise for statistical significance, it may also be seen as a limitation, restricting the generalisability of the results to other population groups like older adults, elite athletes, females, and those with an increased BMI. These are all aspects that may impact metabolism, and therefore free radical production and the induced oxidative stress. It is important that these factors be considered by researchers when planning to use this model.

## 6. Conclusions

The model we describe used a simple exercise regime on a Wattbike, which is readily available at many sport/exercise laboratories as well as fitness gyms/centres and is widely used by athletes, coaches, and researchers as a tool for cycling performance assessment. The controlled exercise regime employed reliably and repeatably induced mild oxidative stress in human participants. This was achieved in an environment that has a low likelihood of causing adverse events given that participants are allowed to exercise to a voluntary exhaustion point rather than given a set number of repetitions or sprints to perform. The use of the rooibos intervention did not induce any complications in the generation of oxidative stress, nor did it cause any adverse reactions in the study participants. This shows that such interventions can be successfully introduced along with the exercise-induced oxidative stress model. It is important to note that the level of the rooibos intervention did not equate to a “mega-dose” intake, but rather was equivalent to six cups of fermented rooibos herbal tea, previously shown to be safe when consumed daily [25]. In our study, the rooibos intervention was able to modulate certain oxidative stress markers, showing that the antioxidant and related bioactivities of herbal infusions can be successfully tested in this exercise-induced oxidative stress model. We thus propose that this is a viable model for the study of mild oxidative stress in human participants. Future research could be conducted on an increased number of participants and then also consider the level of physical fitness of the individual participants, as the ergogenic effect of these herbal interventions may depend on the fitness level/training status of the study participants.

## Figures and Tables

**Table 1:** Antioxidant content and capacity of the study beverages as determined by HPLC and spectrophotometric analysis.

Parameter	Fermented Rooibos Beverage (375 mL)	Placebo Beverage (375 mL)
Flavonols (mg QE)	93.7 ± (1.67)	11.27 ± 5.03
Flavanols (mg CE)	37.6 ± 3.67	0
Total polyphenolic content (mg GAE)	333 ± 1.72	0
FRAP (µmol AAE)	1701 ± 4.55	52.55 ± 2.86
TEAC (µmol TE)	2118 ± 19.3	0
Aspalathin (mg)	8.37 ± 0.04	0
Orientin (mg)	6.69 ± 0.03	0
Isoorientin (mg)	15.50 ± 0.17	0
Isovitexin (mg)	2.52 ± 0.05	0
Vitexin (mg)	1.95 ± 0.03	0
Hyperroside (mg)	7.26 ± 0.05	0
Quercetin (mg)	2.37 ± 0.02	0
Luteolin (mg)	0.57 ± 0.02	0
Chrysoeriol (mg)	0.11 ± 0.00	0

Values in columns represent the means ± standard deviations (*n* = 3). Abbreviations: FRAP = ferric reducing antioxidant power; TEAC = Trolox equivalent antioxidant capacity; QE = quercetin equivalents; CE = catechin equivalents; GAE = gallic acid equivalents; AAE = ascorbic acid equivalents; TE = Trolox equivalents.

**Table 2:** Anthropometric characteristics and other health indicators of the final study population (*n* = 30).

Variable	Placebo Session	Rooibos Session	*p* Value
Age	26.23 (6.53)	26.23 (6.53)	1.0
Mass (kg)	73.45 (14.31)	73.45 (14.31)	1.0
Height (m)	1.73 (0.08)	1.73 (0.08)	1.0
Waist circumference (cm)	102 (8.38)	102 (8.38)	1.0
BMI (kg/m)	24.45 (4.22)	24.45 (4.22)	1.0
SBP (mm Hg)	125.4 (11.57)	124.5 (10.59)	0.563
DBP (mm Hg)	73.33 (7.76)	73.80 (8.23)	0.573
Hb (g/dL)	15.28 (1.23)	15.21 (1.08)	0.676
Glucose (mmol/L)	4.91 (0.23)	4.97 (0.33)	0.359
Total cholesterol (mmol/L)	4.51 (0.10)	4.56 (0.15)	0.979

Values in columns are expressed as mean and standard deviation (SD) (*n* = 30). Abbreviations: BMI = body mass index; DBP = diastolic blood pressure; SBP = systolic blood pressure; Hb = haemoglobin.

**Table 3:** Effects of the two intervention beverages on the blood glutathione and its redox status in the study participants.

Marker	Group	0 h		1.5 h		IAE		1 h Rest		24 h Rest		*p* Value			0–1.5 h	0 h-IAE	0–1 h Rest	0–24 h Rest
		Mean	SD	Mean	SD	Mean	SD	Mean	SD	Mean	SD	Time	Time x Group	Group	%∆	%∆	%∆	%∆
tGSH [µmol/L]	Placebo	1330	800	1370	878	1643	1033	1489	827	1327	800	<0.001	0.055	0.617	3%	24%	12%	0%
	Rooibos	1313	855	1603	1010	1708	1170	1446	893	1648	983				22%	30%	10%	26%
GSH [µmol/L]	Placebo	1306	816	1344	895	1615	1049	1470	836	1314	806	<0.001	0.063	0.604	3%	24%	13%	1%
	Rooibos	1300	860	1580	1019	1686	1180	1431	899	1637	986				22%	30%	10%	26%
GSSG [µmol/L]	Placebo	11.97	20.67	12.74	24.46	14.22	24.03	9.42	10.75	6.34	6.71	0.003	0.436	0.427	6%	19%	−21%	−47%
	Rooibos	6.58	5.68	11.71	13.57	10.58	12.28	7.27	7.59	5.16	4.03				78%	61%	11%	−22%
GSH/GSSG Ratio	Placebo	309	264	343	352	383	491	393	407	424	380	0.003	0.429	0.816	11%	24%	27%	37%
	Rooibos	380	476	327	349	396	416	377	359	484	477				−14%	4%	−1%	27%

Values in columns are expressed as mean and standard deviation (SD) (*n* = 30). Abbreviations: tGSH = total reduced glutathione; GSSG = oxidised glutathione, GSH = reduced glutathione; 0 h = time zero hour; 1.5 h = 90 min after consumption of the intervention beverage and snack; IAE = time immediately after exercise completed; 1 h rest = 60 min of rest after completion of exercise; and 24 h = 24 h after completion of the exercise regime. A repeated-measures analysis of variance (RM-ANOVA) was used for statistical analysis. A *p*-value < 0.05 was considered significant.

**Table 4:** Effects of the two intervention beverages on antioxidant, oxidative lipid, and protein damage biomarkers.

Marker	Group	0 h		1.5 h		IAE		1 h Rest		24 h Rest		*p* Value			0–1.5 h	0 h-IAE	0–1 h Rest	0–24 h Rest
		Mean	SD	Mean	SD	Mean	SD	Mean	SD	Mean	SD	Time	Time x Group	Group	%∆	%∆	%∆	%∆
CD [µmol/L]	Placebo	15.41	3.44	15.01	3.41	15.91	3.18	15.71	3.77	15.44	3.67	0.051	0.374	0.962	−3%	3%	2%	0%
	Rooibos	15.33	3.81	15.25	3.41	15.63	3.65	15.30	3.38	15.77	3.39				−1%	2%	0%	3%
TBARS [µmol/L]	Placebo	10.56	1.28	10.28	1.32	10.17	1.27	10.37	1.29	10.70	1.11	0.004	0.876	0.689	−3%	−4%	−2%	1%
	Rooibos	10.89	1.33	10.25	1.31	10.20	1.09	10.36	1.30	10.81	1.14				−6%	−6%	−5%	−1%
PC [µmol/L]	Placebo	2.30	0.60	2.26	0.77	2.33	0.70	2.40	0.67	2.48	1.00	0.728	0.144	0.760	−2%	1%	4%	8%
	Rooibos	2.36	0.64	2.61	0.59	2.43	0.72	2.24	0.58	2.35	0.83				11%	3%	−5%	0%
UCB [µmol/L]	Placebo	5.53	2.89	5.65	3.34	5.90	3.79	5.49	3.16	5.10	3.43	0.002	0.206	0.996	2%	7%	−1%	−8%
	Rooibos	5.04	3.07	5.56	2.76	6.41	3.36	5.30	2.88	5.39	3.50				10%	27%	5%	7%
FRAP [µmol/L]	Placebo	1020	161	1021	140	1055	172	1169	179	1006	132	<0.001	0.463	0.881	0%	3%	15%	−1%
	Rooibos	1010	173	1021	227	1028	228	1207	201	1034	158				1%	2%	20%	2%

Values in columns are expressed as mean and standard deviation (SD) (*n* = 30). Abbreviations: TBARS = thiobarbituric acid reactive substance; CDs = conjugated dienes; PC = protein carbonyl. 0 h = time zero hour; 1.5 h = 90 min after consumption of the intervention beverage and snack; IAE = time immediately after exercise completed; 1 h rest = 60 min of rest after completion of exercise; and 24 h = 24 h after completion of the exercise regime. A repeated-measures analysis of variance (RM-ANOVA) was used for statistical analysis. A *p*-value < 0.05 was considered significant. While the time effect shows whether there was a significant difference between the 5 measured time points, the group effect shows whether there was an effect between the rooibos and placebo groups. The time x group effect shows whether there was an interaction between the two effects.

**Table 5:** Effects of intervention beverages on DNA damage.

Marker	Group	0 h		1.5 h		IAE		1 h Rest		24 h Rest		*p* Value			0–1.5 h	0 h-IAE	0–1 h Rest	0–24 h Rest
		Mean	SD	Mean	SD	Mean	SD	Mean	SD	Mean	SD	Time	Time x Group	Group	%∆	%∆	%∆	%∆
Lysis [% tail DNA]	Placebo	23.92	8.28	26.60	7.75	27.15	7.41	26.27	7.38	26.39	7.23	0.011	0.228	0.314	11%	13%	10%	10%
	Rooibos	26.63	8.42	27.79	7.46	28.34	7.53	29.71	7.27	26.45	7.17				4%	6%	12%	−1%
H_2_O_2_ [% tail DNA]	Placebo	26.79	8.29	30.46	8.62	31.80	7.88	32.00	7.06	31.65	8.10	<0.001	0.519	0.176	14%	19%	19%	18%
	Rooibos	31.00	11.53	32.26	8.39	33.52	8.63	35.55	9.76	33.78	9.04				4%	8%	15%	9%
FPG [% tail DNA]	Placebo	5.42	4.51	7.30	3.79	6.93	3.52	8.29	4.67	9.07	4.90	<0.001	0.956	0.667	35%	28%	53%	67%
	Rooibos	5.50	4.37	8.09	4.83	6.90	5.24	8.80	4.61	9.46	4.45				47%	25%	60%	72%

Values in columns are expressed as mean and standard deviation (SD) (*n* = 30). Abbreviations: FPG = formamidopyrimidine DNA glycosylase; 0 h = time zero hour; 1.5 h = 90 min after consumption of the intervention beverage and snack; IAE = time immediately after exercise completed; 1 h rest = 60 min of rest after completion of exercise; and 24 h = 24 h after completion of the exercise regime. A repeated-measures analysis of variance (RM-ANOVA) was used for statistical analysis. A *p*-value < 0.05 was considered significant. While the time effect shows whether there was a significant difference between the 5 measured time points, the group effect shows whether there was an effect between the rooibos and placebo groups. The time x group effect shows whether there was an interaction between the two effects.

**Table 6:** Effects of the two intervention beverages on participants’ serum chemistry.

Marker	Group	0 h		1.5 h		IAE		1 h Rest		24 H Rest		*p* Value			0−1.5 h	0 h-IAE	0−1 h Rest	0−24 h Rest
		Mean	SD	Mean	SD	Mean	SD	Mean	SD	Mean	SD	Time	Time x Group	Group	%∆	%∆	%∆	%∆
AST [U/L]	Placebo	31.16	15.44	31.48	14.13	33.79	16.23	30.92	14.07	30.11	13.08	0.016	0.275	0.167	1%	8%	−1%	−3%
	Rooibos	27.22	8.58	26.15	7.29	28.94	7.54	26.55	6.65	28.02	11.80				−4%	6%	−2%	3%
ALT [U/L]	Placebo	25.07	20.08	24.54	18.71	26.13	22.38	24.65	19.78	25.20	19.13	0.2	0.288	0.261	−2%	4%	−2%	1%
	Rooibos	18.41	10.31	19.38	11.43	20.26	11.50	20.25	10.91	22.77	23.28				5%	10%	10%	24%
ALP [U/L]	Placebo	77.28	23.78	77.21	21.38	82.54	25.21	75.90	21.71	75.83	22.60	<0.001	0.200	0.903	0%	7%	−2%	−2%
	Rooibos	73.74	21.36	75.42	21.85	83.28	24.68	77.00	23.30	75.84	20.90				2%	13%	4%	3%
GGT [U/L]	Placebo	27.47	12.93	26.63	11.55	28.24	12.54	27.02	12.23	26.77	12.36	<0.001	0.303	0.809	−3%	3%	−2%	−3%
	Rooibos	25.91	12.37	26.30	12.38	28.00	13.11	26.60	13.06	25.46	11.97				2%	8%	3%	−2%
TBIL [µmol/L]	Placebo	12.74	6.58	12.15	6.56	13.42	7.19	12.19	6.66	13.30	7.41	0.338	0.382	0.691	−5%	5%	−4%	4%
	Rooibos	12.71	6.81	14.81	13.55	14.03	6.61	12.25	5.81	13.35	6.94				17%	10%	−4%	5%
DBIL [µmol/L]	Placebo	2.29	0.75	2.11	0.87	2.23	1.04	2.14	0.81	2.30	0.89	0.025	0.438	0.918	−8%	−2%	−7%	1%
	Rooibos	2.13	0.81	2.17	0.76	2.25	0.86	2.09	0.77	2.33	0.88				2%	6%	−2%	10%
TP [g/L]	Placebo	80.54	7.12	81.02	7.58	86.83	10.69	81.00	7.29	81.63	7.73	<0.001	0.582	0.793	1%	8%	1%	1%
	Rooibos	81.30	7.30	81.72	7.69	87.60	8.60	82.03	6.69	80.83	7.15				1%	8%	1%	−1%
ALB [g/L]	Placebo	51.13	3.26	51.73	3.33	54.27	5.48	51.65	2.64	50.45	7.29	<0.001	0.599	0.822	1%	6%	1%	−1%
	Rooibos	50.52	7.74	51.35	3.24	54.23	5.64	52.47	4.39	51.68	2.96				2%	7%	4%	2%
BUN [mmol/L]	Placebo	4.58	1.19	4.57	1.09	4.67	1.03	4.92	1.10	4.63	1.01	<0.001	0.503	0.985	0%	2%	7%	1%
	Rooibos	4.42	1.24	4.52	1.10	4.69	1.11	4.99	1.26	4.72	0.96				2%	6%	13%	7%
CREAT [µmol/L]	Placebo	86.35	19.21	89.97	16.92	113.17	99.48	90.82	21.46	84.07	22.70	0.069	0.285	0.816	4%	31%	5%	−3%
	Rooibos	86.77	24.06	90.92	26.70	95.93	21.43	94.73	22.58	88.60	19.15				5%	11%	9%	2%
UA [µmol/L]	Placebo	358.98	73.96	371.25	77.36	370.52	80.29	497.28	104.51	376.83	77.51	<0.001	0.399	0.765	3%	3%	39%	5%
	Rooibos	360.42	78.98	364.22	75.77	369.60	85.27	512.98	110.11	396.43	74.65				1%	3%	42%	10%
GLUC [nmol/L]	Placebo	5.08	0.54	4.65	0.69	4.85	1.02	5.02	0.88	5.12	0.46	<0.001	0.466	0.518	−9%	−5%	−1%	1%
	Rooibos	5.19	0.35	4.55	0.68	5.03	0.98	4.91	0.80	5.37	0.60				−12%	−3%	−5%	4%
LDH [U/L]	Placebo	184.87	47.65	188.84	40.23	203.00	53.38	190.00	44.15	193.22	48.20	0.063	0.464	0.569	2%	10%	3%	5%
	Rooibos	188.07	47.93	180.37	44.06	191.71	32.61	186.71	40.28	184.83	35.19				−4%	2%	−1%	−2%
CK [U/L]	Placebo	510.21	567.18	528.27	577.61	552.40	643.99	510.96	579.06	450.04	453.91	0.283	0.172	0.149	4%	8%	0%	−12%
	Rooibos	390.67	401.41	312.28	241.13	356.57	259.29	327.76	237.30	355.02	268.70				−20%	−9%	−16%	−9%
LAC [U/L]	Placebo	2.80	1.41	2.92	1.09	12.43	2.98	3.21	1.42	2.32	0.78	<0.001	0.461	0.363	4%	344%	15%	−17%
	Rooibos	2.76	1.17	3.00	1.25	13.27	3.03	3.44	1.14	2.54	1.33				9%	381%	25%	−8%

Values in columns are expressed as mean and standard deviation (SD) (*n* = 30). Abbreviations: ALB = albumin; ALP = alkaline phosphatase; AST = aspartate aminotransferase; ALT = alanine aminotransferase; BUN = blood urea nitrogen; CK = creatine kinase; CREAT = creatinine; DBIL = direct bilirubin; GLUC = glucose; GGT = gamma glutamyl transferase; LAC = lactate; LDH = lactate dehydrogenase; TBIL = total bilirubin; TP = total protein, UA = uric acid; 0 h = time zero hour; 1.5 h = 90 min after consumption of the intervention beverage and snack; IAE = time immediately after exercise completed; 1 h rest = 60 min of rest after completion of exercise; and 24 h = 24 h after completion of the exercise regime. A repeated-measures analysis of variance (RM-ANOVA) was used for statistical analysis. A *p*-value < 0.05 was considered significant. While the time effect shows whether there was a significant difference between the 5 measured time points, the group effect shows whether there was an effect between the rooibos and placebo groups. The time x group effect shows whether there was an interaction between the two effects.

## Data Availability

The original contributions presented in this study are included in the article/Supplementary Material. Further inquiries can be directed to the corresponding author.

## Supplementary Materials

The following supporting information can be downloaded at https://www.mdpi.com/article/10.3390/nu17182971/s1, File S1. CONSORT Checklist and HPLC chromatograms of study beverages (Reference [47] is cited in the supplementary materials).

## Abbreviations

The following abbreviations are used in this manuscript:

AAE	Ascorbic acid equivalents
ALB	Albumin
ALP	Alkaline phosphatase
ALT	Alanine aminotransferase
AST	Aspartate aminotransferase
BHT	Butylated hydroxytoluene
BMI	Body mass index
BSA	Bovine serum albumin
BUN	Blood urea nitrogen
CD	Conjugated dienes
CE	Catechin equivalents
CK	Creatine kinase
CREAT	Creatinine
DBIL	Direct bilirubin
DBP	Diastolic blood pressure
DDW	Double-distilled water
DNPH	Dinitrophenylhydrazine
DTNB	5,5′-dithiobis-2-nitrobenzoic acid
EDTA	Ethylenediaminetetraacetic acid
FPG	Formamidopyrimidine DNA glycosylase
FRAP	Ferric reducing antioxidant power
GAE	Gallic acid equivalents
GGT	Gamma glutamyl transferase
GLUC	Glucose
GR	Glutathione reductase
GSH	Reduced glutathione
GSSG	Oxidised glutathione
H_2_O_2_	Hydrogen peroxide
Hb	Haemoglobin
HPLC	High-performance liquid chromatography
LAC	Lactate
LDH	Lactate dehydrogenase
M2VP	Methyl-2-vinylpyridinium
MDA	Malondialdehyde
MPA	Meta-phosphoric acid
NADPH	Nicotinamide adenine dinucleotide phosphate hydrogen
PC	Protein carbonyls
QE	Quercetin equivalents
RPE	Rate of perceived exertion
RT	Room temperature
SBP	Systolic blood pressure
SST	Serum separates
TBARS	Thiobarbituric acid reactive substances
TBIL	Total bilirubin
TCA	Trichloro acetic acid
TE	Trolox equivalents
TEAC	Trolox equivalent antioxidant capacity
tGSH	Total reduced glutathione
TP	Total protein
UA	Uric acid
UCB	Unconjugated bilirubin
W	Watts
